# UPLC-MS/MS profiling, antioxidant and hemorheological effects of D101 resin-based purified flavonoids from *Allium polyrhizum*

**DOI:** 10.3389/fphar.2026.1755360

**Published:** 2026-06-03

**Authors:** Caiquan Zhao, Lige Bai, Jian Gao, Jie Liu, Ling Lu, Meng Wang, Jiahua Guo, Peng Zhao

**Affiliations:** College of Ecology and Environment, Baotou Teachers’ College, Baotou, China

**Keywords:** *Allium polyrhizum Turcz.* ex Regel, antioxidant, blood stasis, flavonoids, macroporous resins

## Abstract

**Introduction:**

Oxidative stress and blood stasis represent critical pathological drivers underlying the progression of cardiovascular and cerebrovascular diseases. Total flavonoids derived from *Allium polyrhizum* Turcz. ex Regel (APTF) have been reported to possess potential antioxidant and blood-activating properties. However, the purification process and systematic pharmacological characteristics of APTF remain insufficiently elucidated, which substantially limits its further development and practical application.

**Methods:**

In this study, orthogonal experiments were performed to optimize the purification conditions of APTF using D101 macroporous resin. Chemic antioxidant activity of purified APTF was evaluated via multiple free radical scavenging assays. A rat model of blood stasis was established to verify the *in vivo* efficacy of APTF, with hemorheological, vascular endothelial, and blood coagulation indices determined for comprehensive assessment. Additionally, ultra-high performance liquid chromatography coupled with tandem mass spectrometry (UPLC-MS/MS) was employed to systematically identify the metabolic components of APTF.

**Results:**

The optimized purification protocol yielded a high APTF recovery rate of 95.112±1.507%. Purified APTF exhibited potent free radical scavenging activity. *In vivo* experimental results demonstrated that APTF effectively ameliorated hemorheological abnormalities, restored vascular endothelial functional homeostasis, and exerted remarkable anticoagulant effects in blood stasis model rats. A total of 309 flavonoid metabolites were successfully identified, which constituted the potential material basis for the pharmacological activities of APTF.

**Discussion:**

This study established a stable and reliable purification protocol for APTF using D101 macroporous resin. The purified APTF displays prominent antioxidant and blood-activating bioactivities, conferring great application potential for the development of vascular protective functional products and therapeutic agents. Future investigations will focus on screening the active monomer components of APTF and clarifying its underlying molecular mechanisms of action.

## Introduction

Antioxidants are critical for maintaining human health by alleviating oxidative stress and preventing cellular damage caused by excessive free radicals. Uncontrolled free radicals can induce cell dysfunction, gene mutations, and various diseases (Lobo et al., 2010). As electron donors, antioxidants stabilize free radicals and protect cells and tissues from oxidative injury (Valko et al., 2007). Natural antioxidants mainly include vitamins, minerals, and phytochemicals abundant in plants. Blood stasis is a core pathological concept in traditional Chinese medicine, first documented in Huangdi Neijing (Li et al., 2014). Modern pharmacological studies have shown that several Chinese botanical drugs, such as *Salvia miltiorrhiza* Bunge, *Cynanchum otophyllum* C. K. Schneid, and *Angelicas sinensis* (Oliv.) Diels, can alleviate blood stasis by improving hemorheology, inhibiting thrombosis, reducing inflammation, and regulating vascular endothelial factors (Du et al., 2010; Jin et al., 2008; Liu et al., 2014).

*Allium polyrhizum Turcz.* ex Regel (Liliaceae) is a medicinal plant distributed in northern and northwestern China. It is commonly used as a seasoning, and also serves as a traditional Mongolian folk medicine for detoxification, anti-inflammation and stomach regulation. Flavonoids from *Allium polyrhizum* (APTF) exert anti-inflammatory, antitumor, antioxidant, and anti-aging effects with high safety (Li et al., 2025; Martinez-Coria et al., 2023; Tao et al., 2026). Since flavonoids cannot be synthesized endogenously in humans, there is growing demand for flavonoid-enriched functional foods with high purity. Macroporous resin is widely applied in the purification of flavonoids due to its low cost, simple operation, high adsorption capacity, and suitability for industrial production (Shen et al., 2022a; Beeler et al., 2025). However, studies on the purification, antioxidant capacity, and blood stasis-resolving effects of APTF remain insufficient.

In this study, APTF was separated and purified by D101 macroporous resin, with the optimal purification parameters systematically determined. UPLC-MS/MS was employed for the profiling and identification of the chemical metabolites in APTF. The chemic antioxidant activity of APTF and its *in vivo* hemorheological effects were assessed. Collectively, these findings provide a solid theoretical foundation for the development of vascular-protective products derived from this medicinal and edible dual-purpose plant.

## Materials and methods

### Study objects

The crude APTF used in this study is stored in the laboratory. The experimental Wistar rats were purchased from Sibeifu (Beijing) Biotechnology Co., Ltd., aged 6–8 weeks, weighing 250–350 g. The animals were acclimatized for 1 week in an environment with standardized room temperature (22 ± 2) °C, relative humidity (55 ± 5)%, and 12 h light/dark cycle, with free access to food and water.

### Blood stasis animal model

Forty-eight Wistar rats with balanced gender distribution, were acclimatized for 1 week and randomly divided into six groups according to body weight (n = 8 per group): control group, blood stasis model group, aspirin group (60 mg/kg), and low-, medium- and high-dose APTF groups. All rats were orally administered once daily for seven consecutive days. The control and model groups received an equal volume of distilled water. On day 7, one hour after administration, all groups except the control group were subcutaneously injected with adrenaline (8 μg/kg) twice at 4-h intervals. Rats were immersed in ice water for 5 min between the two adrenaline injections. After treatment, food was deprived and water was provided *ad libitum* until testing the next morning.

### Screening of macroporous resin

Pretreated HPD500, AB-8, D101, NKA-9, H103, and D4020 resins (2.0 g each) were placed in 150-mL flasks with 20 mL of APTF crude extract (0.262 mg/mL). After shaking at 25 °C and 120 r/min for 24 h, the filtrate was collected to determine equilibrium flavonoid concentration, and adsorption capacity and adsorption rate were calculated. Resins were rinsed, dried, and eluted with 20 mL of 95% ethanol–water mixture for another 24 h, and the desorption rate was determined.

$$\text{Calculations }Q=\frac{\left(C_{o}-C_{1}\right)v}{m},A\left(\%\right)=\frac{C_{0}-C_{1}}{C_{0}}\times 100,D\left(\%\right)=\frac{C_{2}}{C_{0}-C_{1}}\times 100$$

*Q* = adsorption capacity (mg/g);

*A* = adsorption rate (%);

*D* = desorption rate (%);

*C*_*0*_ = initial flavonoid concentration (mg/mL);

*C*_*1*_ = equilibrium supernatant concentration (mg/mL);

*C*_*2*_ = desorption equilibrium concentration (mg/mL);

*V* = extract volume (mL);

*m* = resin mass (g).

### Static and dynamic tests of D101 macroporous adsorption resin

Static adsorption and desorption: 2.0 g of D101 resin was accurately weighed and mixed with 20 mL of APTF crude extract (0.262 mg/mL). The flavonoid concentration in the supernatant were determined at 0.5, 1, 2, 4, 6, 8, 12 and 24 h during shaking adsorption/desorption, and adsorption/desorption rates were calculated. Effect of sample concentration: 2.0 g D101 resin was placed in a 150 mL conical flask with 20 mL APTF extracts at 0.103, 0.312, 0.525, 0.692, 0.918 and 1.094 mg/mL. Static tests were performed as above to calculate adsorption/desorption rates. Effect of temperature: 2.0 g D101 resin was mixed with 20 mL APTF extract (0.715 mg/mL) and shaken in water baths at 25, 30, 35, 40 °C and 45 °C. Adsorption and desorption rates were determined using the same procedure.

Determination of loading and elution volumes: 5 g of pretreated D101 resin was wet-packed into a 2 × 30 cm column (with a bed height of 8 cm). With the sample concentration set at 0.734 mg/mL and flow rate of 2 mL/min, effluent flavonoid content was measured every 15 min. Leakage point was defined as reached 10% of the initial sample concentration, and the corresponding volume was the equilibrium loading volume. The column was washed with distilled water (2 mL/min) until the reactions yielded negative results, then eluted with 70% aqueous ethanol (2 mL/min) until no flavonoids were detected. Single-factor experiments: Dynamic adsorptionand desorption tests were conducted to investigate loading flow rate (2–6 mL/min), sample pH (3–7), ethanol concentration (10%–95%) and elution flow rate (2–6 mL/min). Dynamic elution curves were plotted, and adsorption rate, desorption rate and recovery rate were calculated.

$$\text{The recovery rate formula}:R\left(\%\right)=\frac{C_{2}V_{2}}{C_{0}V_{0}}\times 100$$

where R = total flavonoid recovery (%), C_0_ = initial flavonoid concentration (mg/mL), V_0_ = sample volume (mL), C_2_ = eluent flavonoid concentration (mg/mL), V_2_ = eluent volume (mL). Orthogonal optimization: Based on single-factor results, orthogonal experiments were performed with elution flow rate, sample pH and ethanol concentration as factors, and flavonoid recovery as the index. Factors and levels are listed in Supplementary Table S1.

### Chemic antioxidant activity determination

Crude extract and purified product of APTF were accurately weighed, dissolved in 100 mL of distilled water, and serially diluted to obtain gradient solutions (0.2, 0.4, 0.6, 0.8, 1.0, 1.2 mg/mL). Using vitamin C (Vc) as the positive control, DPPH·, ·OH, O_2_^−^· and ABTS^+^· scavenging abilities were determined by corresponding standard methods, and reducing power was evaluated using the potassium ferricyanide reduction method.

### Hemorheological index measurement

Thirty minutes after the last administration, rats were anesthetized with 2% pentobarbital sodium (i.p.). Abdominal aortic blood was collected, anticoagulated with heparin sodium (500 U/mL), and incubated at room temperature for 20 min. Whole blood viscosity was detected at shear rates of 10/s, 60/s, 120/s and 150/s using an automated blood rheometer. Blood samples anticoagulated with sodium citrate (1:4) were used for erythrocyte sedimentation rate (ESR) and hematocrit (HCT) analysis. After ESR measurement, the samples were centrifuged at 3,000 rpm for 30 min to determine HCT values. Plasma levels of ET, TXA2, PGI2 and NO, as well as APTT, PT, TT and FIB were measured using commercial kits according to the manufacturer’s instructions.

### Sample preparation and extraction

The crude APTF extract was lyophilized using a vacuum freeze dryer (Scientz-100F). The lyophilized sample was ground in a mixer mill (MM 400, Retsch) with a zirconia bead at 30 Hz for 1.5 min. Subsequently, 100 mg of lyophilized powder was dissolved in 1.2 mL of 70% methanol solution, vortexed for 30 s at 30-min intervals for six cycles in total, and stored overnight in a refrigerator at 4 °C. After centrifugation at 12,000 rpm for 10 min, the supernatant was filtered prior to UPLC-MS/MS analysis.

### UPLC conditions

APTF extracts were analyzed by UPLC-ESI-MS/MS. UPLC separation was performed on an Agilent SB-C18 column (1.8 µm, 2.1 mm × 100 mm) using mobile phases consisting of solvent A (0.1% formic acid in water) and solvent B (0.1% formic acid in acetonitrile). Gradient elution was initiated at 95% A and 5% B, linearly changed to 5% A and 95% B within 9 min, held for 1 min, then restored to 95% A and 5% B within 1.10 min and maintained for 2.9 min. The flow rate was 0.35 mL/min, column temperature 40 °C, and injection volume 4 μL. The effluent was introduced into an ESI-triple quadrupole-linear ion trap (QTRAP)-MS system.

### ESI-Q TRAP-MS/MS

LIT and QQQ scans were obtained using an AB4500 Q TRAP UPLC-MS/MS system equipped with an ESI Turbo Ion-Spray interface, operating in positive and negative ion modes and controlled by Analyst 1.6.3 software (AB Sciex). Key ESI source parameters were set as follows: turbo spray source; temperature 550 °C; IS 5500 V (positive)/−4500 V (negative); GS I, GS II, and CUR at 50, 60, and 25.0 psi, respectively; and high collision-activated dissociation. Instrument tuning and mass calibration were conducted with 10 and 100 μmol/L polypropylene glycol solutions in QQQ and LIT modes, respectively. QQQ scans were acquired in MRM mode with medium collision gas (nitrogen), and DP and CE were optimized for individual transitions. MRM transitions were monitored per period based on eluted metabolites.

### Statistical analysis

The experimental results are presented as mean ± standard deviation, and statistical analysis of the experimental data was conducted using Design Expert 12.0 and SPSS 20.0 software.

## Results

### Static experiment of D101 macroporous adsorption resin

**FIGURE 1:**
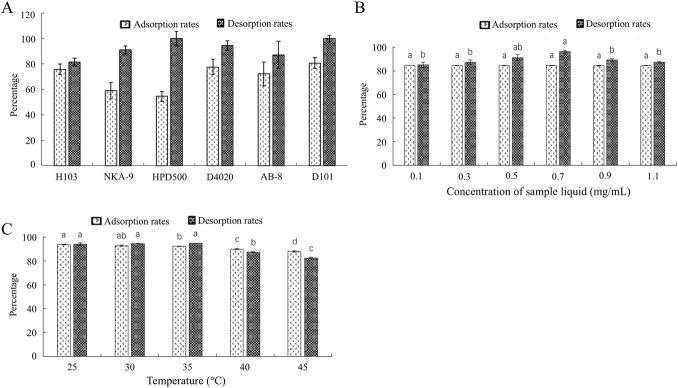
Factors affecting the static adsorption and desorption rates of D101 macroporous resin. The adsorption and desorption rates of HPD500, AB-8, D101, NKA-9, H103, and D4020 macroporous adsorption resins **(A)** The influence of the total flavonoid concentration **(B)** and temperature **(C)** on the adsorption and desorption rates of D101 macroporous resin.

Among the six screened resins, D101 macroporous adsorption resin exhibited the most ideal static adsorption and desorption performance for APTF, with the highest adsorption and desorption rates of 80.57% and 99.89%, respectively (Figure 1A). Its large specific surface area and appropriate pore size were favorable for APTF adsorption–desorption. With increasing sample concentration, the adsorption rate showed no significant difference (*P* > 0.05), whereas the desorption rate first increased and then decreased, peaking at 0.7 mg/mL (*P* < 0.05) (Figure 1B). Elevated temperatures significantly reduced both adsorption and desorption rates (*P* < 0.05) (Figure 1C), indicating that 25 °C–30 °C was optimal for the process. Collectively, these results confirmed that APTF purification with D101 resin was feasible at room temperature.

### Dynamic experiment of D101 macroporous adsorption resin

**FIGURE 2:**
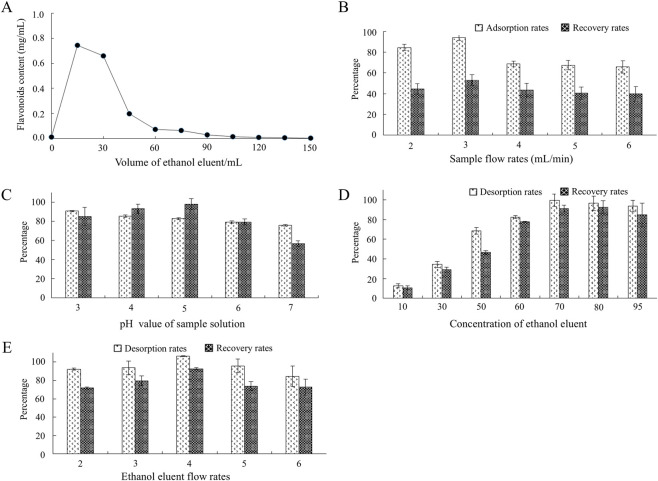
Factors affecting the dynamic adsorption, desorption and recovery rates of D101 macroporous resin. The influence of factors such as ethanol-water mixture eluent volume **(A)** sample flow rate **(B)** sample Ph **(C)** ethanol-water mixture eluent concentration **(D)** and ethanol-water mixture elution flow rate **(E)** on the adsorption, elution, and recovery of APTF by D101 macroporous adsorption resin.

APTF leakage increased with the increase in sample loading volume. The leakage point was reached at a loading volume of 100 mL (effluent flavonoid concentration >10% of the initial sample concentration), which was defined as the equilibrium adsorption volume. Washing with 200 mL of distilled water completely removed water-soluble impurities (Figure 2A). Single-factor optimization showed that the optimal conditions for APTF adsorption, desorption and recovery were: sample flow rate of 3 mL/min (Figure 2B), sample pH 5 (Figure 2C), ethanol eluent concentration 70% (Figure 2D), and elution flow rate 4 mL/min (Figure 2E). Analysis of variance (Supplementary Table S2) confirmed that sample pH, ethanol concentration, and elution flow rate significantly affected the purification of APTF.

Following single-factor optimization, a three-factor and three-level orthogonal design was adopted to further optimize the purification process, with elution flow rate (A), sample pH (B), and ethanol concentration (C) as the experimental factors. Range analysis revealed the order of factor influence for APTF recovery as C > B > A (ethanol concentration > sample pH > elution flow rate). The optimal combination was determined as A_2_B_2_C_3_ (elution flow rate of 4 mL/min, pH 5, 80% ethanol) (Supplementary Table S3). Validation experiments produced a recovery of 95.112% ± 1.507%, with an RSD of 1.59%, indicating good precision of the method. ANOVA showed that these factors had no significant effect on the recovery rate (Supplementary Table S4).

### Determination of chemic antioxidant activity of APTF

**FIGURE 3:**
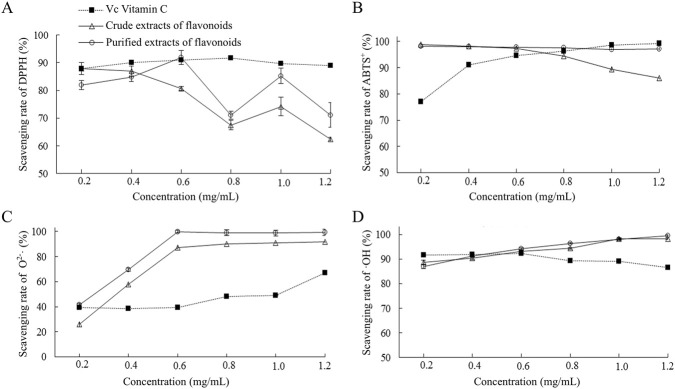
The scavenging effect of APTF on DPPH·, ABTS^+^·, O^2-^·, and ·OH. The scavenging rates of flavonoid crude extracts and purified metabolites at different concentrations on DPPH· **(A)** ABTS^+^· **(B)** O^2-^· **(C)** and ·OH **(D)**.

The chemic antioxidant activity of crude and purified APTF were evaluated. APTF showed decreased DPPH and ABTS^+^· scavenging activities but increased O_2_^−^· and ·OH scavenging with increasing concentration. The crude extract exhibited comparable DPPH scavenging to Vc at ≤ 0.4 mg/mL (*P* > 0.05, Figure 3A) and significantly higher ABTS^+^· scavenging at ≤0.6 mg/mL (*P* < 0.05, Figure 3B). Its O_2_^−^· and ·OH scavenging were similar to those of Vc at low concentrations but superior at high concentrations (*P* < 0.05, Figures 3C,D). Purified APTF displayed stronger antioxidant capacity than the crude extract.

### Effect of APTF on hemorheology

**FIGURE 4:**
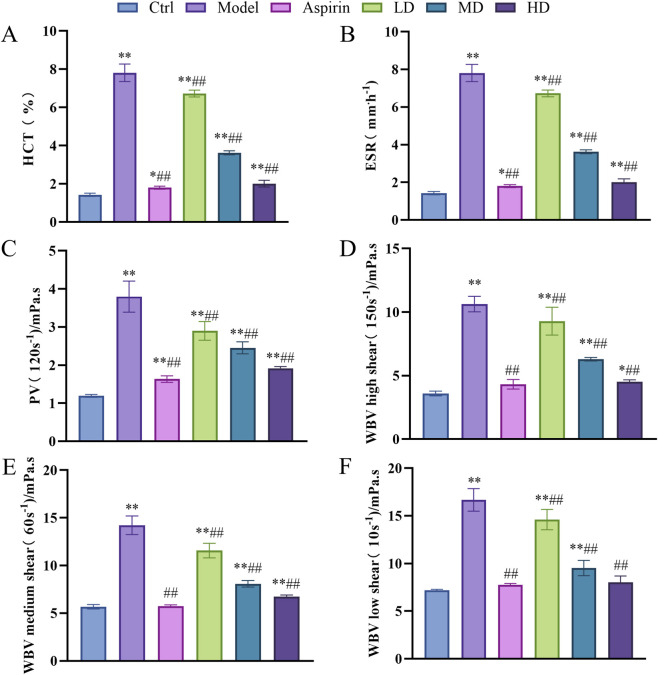
Effect of APTF on the hemorheological parameters of blood stasis rats (x̄±s, n = 8). Relevant parameters of hemorheology analysis, the hematocrit **(A)** erythrocyte sedimentation rate **(B)** plasma viscosity **(C)** and whole blood viscosity (high, medium, and low shear) **(D–F)** Note: * indicates significant difference compared to the normal group (*P* < 0.05), ** indicates extremely significant difference compared to the normal group (*P* < 0.01); # indicates significant difference compared to the model group (*P* < 0.05), ## indicates extremely significant difference compared to the model group (*P* < 0.01), the same below.

To evaluate the blood-activating and stasis-dissolving effects of APTF, hemorheological parameters were assessed in a rat blood stasis model. Compared with the control (Ctrl) group, rats in the model group exhibited significant increases in hematocrit (HCT), erythrocyte sedimentation rate (ESR), plasma viscosity (PV), and whole blood viscosity (WBV) at high, medium, and low shear rates (*P* < 0.01). In contrast, APTF treatment at low (LD), medium (MD), and high (HD) doses significantly reversed these alterations, producing dose-dependent reductions in HCT, ESR, PV, and WBV (*P* < 0.05 or *P* < 0.01) (Figures 4A–F).

### Effect of APTF on vascular endothelial factors

**FIGURE 5:**
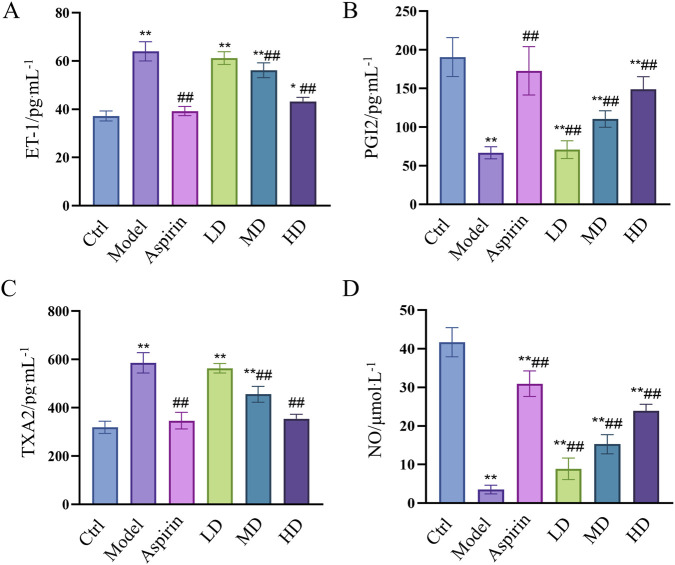
Detection of relevant vascular endothelial factors. Effect of APTF on ET-1 **(A)** PGI2 **(B)** TXA2 **(C)** and NO **(D)** factors in blood stasis rats.

Analysis of vascular endothelial factors revealed that, relative to the negative control group, model rats exhibited significant increases in TXA2 and ET-1 levels (*P* < 0.01) along with marked decreases in PGI2 and NO levels (*P* < 0.01). Compared with the model group, medium- and high-dose APTF treatments significantly reduced TXA2 levels, while low-dose APTF significantly lowered ET-1 levels; all APTF doses markedly increased NO and PGI2 (*P* < 0.01) (Figures 5A–D).

### Effect of APTF on four coagulation indicators

**FIGURE 6:**
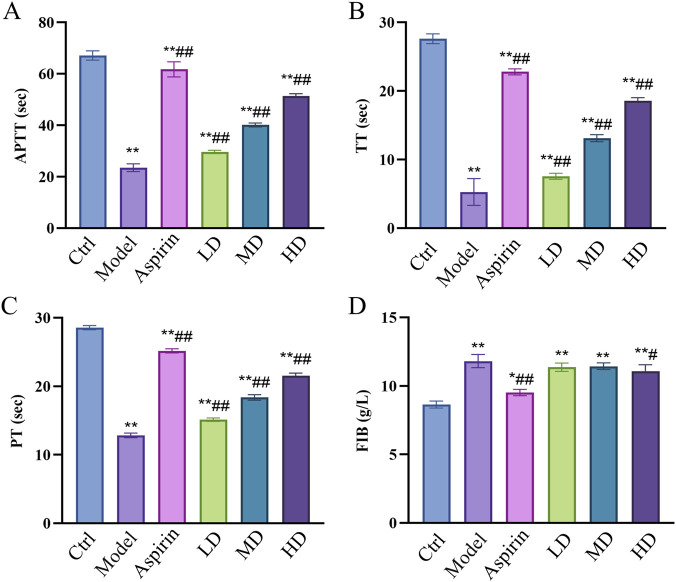
Effect of APTF on four coagulation indicators in blood stasis rats. Coagulation indicators APTT **(A)** TT **(B)** PT **(C)** and FIB **(D)**.

Analysis of plasma coagulation factors showed that, compared with the control group, rats in the model group exhibited significantly shortened APTT, TT and PT (*P* < 0.01) and markedly elevated FIB (*P* < 0.01) (Figures 6A–D). Treatment with low-, medium- and high-dose APTF significantly prolonged APTT, TT and PT and reduced FIB relative to the model group (all *P* < 0.01), although these parameters remained lower than those in the aspirin group. APTF at all tested doses significantly prolonged APTT, TT and PT in acute blood stasis rats, demonstrating obvious anticoagulant capacity.

### Determination of chemical metabolites of APTF

**FIGURE 7:**
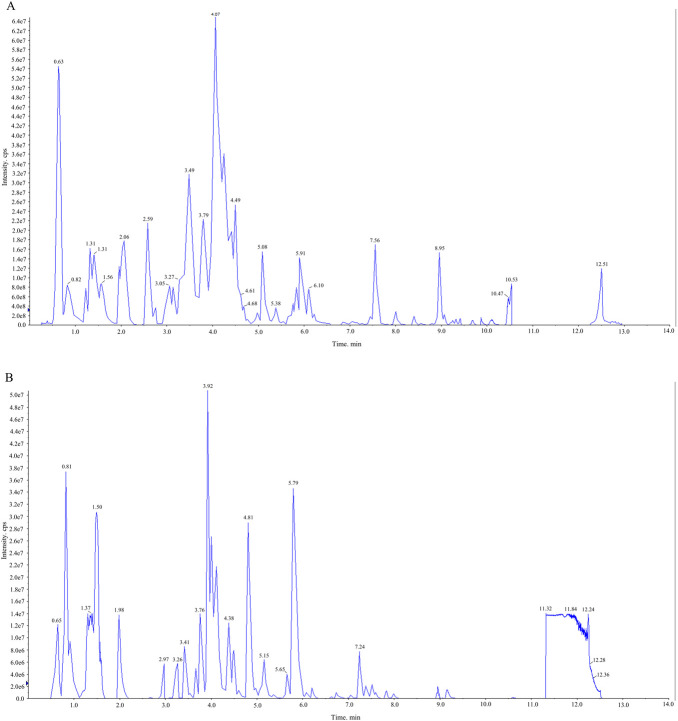
Total ion current of mixed samples in mass spectrometry analysis. Positive ion mode **(A)** Negative ion mode **(B)**.

To determine the chemical composition of APTF, a total of 309 bioactive metabolites were identified by UPLC-MS/MS (Figures 7A,B; Supplementary Table S5). These metabolites included flavonoids, flavonols, dihydroflavonols, anthocyanins, isoflavones, flavonoid C-glycosides, chalcones, dihydroflavonoids, and flavanols; flavonoids accounted for 70.433% of the total metabolites.

## Discussion

Oxidative stress and blood stasis syndrome are core pathological mechanisms underlying cardiovascular and cerebrovascular diseases. Natural antioxidants, particularly plant-derived flavonoids, exhibit free radical-scavenging, endothelial-protective and blood circulation-promoting effects, and have become important research targets for the intervention of related diseases (He et al., 2019; Jung et al., 2018). *Allium polyrhizum Turcz.* ex Regel, a typical Mongolian medicinal plant in northern China, possesses high medicinal value. Its total flavonoids (APTF) have lipid-lowering and hypoglycemic activities, while their purification process and pharmacological effects remain unclear. D101 macroporous resin was selected (adsorption rate 80.57%, desorption rate 99.89%) owing to its favorable pore structure. Static and dynamic tests confirmed room temperature (25 °C–30 °C), a loading volume of 100 mL and 200 mL distilled water as optimal conditions. Orthogonal experiments indicated that ethanol concentration exerted the most prominent effect on the recovery rate. The optimized purification conditions (elution flow rate: 4 mL/min, pH 5, 80% ethanol) yielded a recovery of 95.112% ± 1.507% with acceptable repeatability, demonstrating good stability and feasibility of the procedure.

Chemic antioxidant activity assays showed that APTF scavenged DPPH·, ABTS^+^·, O_2_^−^· and ·OH; crude APTF had comparable or superior scavenging capacity to Vc, and purified APTF had enhanced capacity. These results were consistent with previous reports that plant flavonoids exert potent antioxidant and anti-inflammatory effects by neutralizing free radicals and regulating oxidative stress-related signaling pathways (Shen et al., 2022b; Al-Khayri et al., 2022). Flavonoids from *Carthamus tinctorius* L., *Curcuma longa* L. and other medicinal plants have been verified to exert antioxidant capacity via inhibiting ROS production, activating the SIRT1 and Nrf2 pathways, and regulating NF-κB and apoptosis-related proteins (Chen et al., 2022). Furthermore, research has confirmed that *Curcuma longa* L. and *Astragali Radix* stem and leaf flavonoid extracts both have effective antioxidant capabilities in scavenging reactive oxygen species (Cui et al., 2022; Muflihah et al., 2021). Moreover, leaf extracts of *Ziziphus mauritiana* Lam. possessed the highest antioxidant capacity due to their high contents of bioactive metabolites such as flavonoids and tannins high bioactive metabolites content (Yahia et al., 2020). The above findings collectively demonstrate that flavonoid-rich plant extracts possess potent antioxidant properties.

In the rat blood stasis model, APTF dose-dependently ameliorated hemorheological indices by reducing whole blood viscosity, plasma viscosity, hematocrit and erythrocyte sedimentation rate, which is consistent with findings for other plant flavonoids (He et al., 2023). Further detection showed that APTF downregulated the overproduced ET-1 and TXA2, and upregulated the depleted NO and PGI2, restoring the balance between vasoconstrictors and vasodilators; endothelial injury disrupts this balance, promoting thrombosis (Xu et al., 2009). In addition, APTF prolonged APTT, TT, PT and reduced fibrinogen, exerting anticoagulant capacity; it improved blood stasis by regulating hemorheology, protecting endothelium and inhibiting hypercoagulability. UPLC-MS/MS identified 309 metabolites (mainly flavonoids), which may be the material basis for its activities. Numerous studies have confirmed that flavonoid metabolites such as quercetin, hesperidin, and apigenin possess endothelial protective effects, inhibit platelet aggregation, dilate blood vessels, and improve microcirculation. These actions may underlie the antioxidant and blood-activating effects of APTF (Sharifi-Rad et al., 2022; Ozorowski et al., 2025).

## Conclusion

In conclusion, these results demonstrate that D101 macroporous resin can be efficiently used for the purification of APTF with a stable and feasible process. APTF exhibits potent chemic antioxidant activity and favorable blood-activating and stasis-resolving effects *in vivo*. These beneficial actions are achieved by improving hemorheological parameters, protecting vascular endothelial function, and suppressing blood hypercoagulability, thereby providing a material and pharmacological basis for the further development and utilization of APTF. However, there are several limitations to this study that should be addressed: (i) The monomeric compounds responsible for antioxidant and blood-activating activities should be further identified; (ii) The molecular mechanism underlying the blood circulation-promoting and stasis-removing effects of APTF should be further elucidated.

## Data Availability

The original contributions presented in the study are included in the article/Supplementary Material, further inquiries can be directed to the corresponding author.

## References

[B1] Al-Khayri J. M. Sahana G. R. Nagella P. Joseph B. V. Alessa F. M. Al-Mssallem M. Q. (2022). Flavonoids as potential anti-inflammatory molecules: a review. Molecules 27, 2901. 10.3390/molecules27092901 35566252 PMC9100260

[B2] Beeler N. Huhn T. Rohn S. Colombi R. (2025). Purification of flavonoids from an aqueous cocoa (Theobroma cocoa L.) extract using macroporous adsorption resins. Molecules 30, 2336. 10.3390/molecules30112336 40509222 PMC12155860

[B3] Chen G. Li C. Zhang L. Yang J. Meng H. Wan H. (2022). Hydroxysafflor yellow A and anhydrosafflor yellow B alleviate ferroptosis and parthanatos in PC12 cells injured by OGD/R. Free Radic. Biol. Med. 179, 1–10. 10.1016/j.freeradbiomed.2021.12.262 34923102

[B4] Cui L. Ma Z. Wang D. Niu Y. (2022). Ultrasound-assisted extraction, optimization, isolation, and antioxidant activity analysis of flavonoids from Astragalus membranaceus stems and leaves. Ultrason. Sonochem 90, 106190. 10.1016/j.ultsonch.2022.106190 36215890 PMC9554832

[B5] Du J. P. Shi D. Z. Li T. C. Xu H. Chen H. (2010). Correlation between blood stasis syndrome and pathological characteristics of coronary artery in patients with coronary heart disease. Zhong Xi Yi Jie He Xue Bao 8, 848–852. 10.3736/jcim20100908] 20836975

[B6] He J. Zeng L. Wei R. Zhong G. Zhu Y. Xu T. (2019). Lagopsis supina exerts its diuretic effect via inhibition of aquaporin-1, 2 and 3 expression in a rat model of traumatic blood stasis. J. Ethnopharmacol. 231, 446–452. 10.1016/j.jep.2018.10.034 30394291

[B7] He Y. Jiang H. Du K. Wang S. Li M. Ma C. (2023). Exploring the mechanism of Taohong Siwu decoction on the treatment of blood deficiency and blood stasis syndrome by gut microbiota combined with metabolomics. Chin. Med. 18, 44. 10.1186/s13020-023-00734-8 37088809 PMC10122815

[B8] Jin X. Shen G. Gao F. Zheng X. Xu X. Shen F. (2008). Traditional Chinese drug ShuXueTong facilitates angiogenesis during wound healing following traumatic brain injury. J. Ethnopharmacol. 117, 473–477. 10.1016/j.jep.2008.02.033 18417308

[B9] Jung J. Ko M. M. Lee J. A. Lee M. S. (2018). Recognition of association between blood stasis syndrome and traumatic injury among doctors of Korean medicine: a cross-sectional observation study. Chin. J. Integr. Med. 24, 254–259. 10.1007/s11655-017-2788-y 29327121

[B10] Li S. M. Xu H. Chen K. J. (2014). The diagnostic criteria of blood-stasis syndrome: considerations for standardization of pattern identification. Chin. J. Integr. Med. 20, 483–489. 10.1007/s11655-014-1803-9 24610412

[B11] Li W. Y. Zhang L. Xie X. W. Shi X. F. Ma Q. H. (2025). Advancements in research on the anti-aging effects and mechanisms of flavonoids in natural products. Front. Med. (Lausanne) 12, 1637992. 10.3389/fmed.2025.1637992 40904361 PMC12403997

[B12] Liu P. Duan J. A. Bai G. Su S. L. (2014). Network pharmacology study on major active compounds of siwu decoction analogous formulae for treating primary dysmenorrhea of gynecology blood stasis syndrome. Zhongguo Zhong Yao Za Zhi 39, 113–120. 10.4268/cjcmm20140122 24754179

[B13] Lobo V. Patil A. Phatak A. Chandra N. (2010). Free radicals, antioxidants and functional foods: impact on human health. Pharmacogn. Rev. 4, 118–126. 10.4103/0973-7847.70902 22228951 PMC3249911

[B14] Martinez-Coria H. Arrieta-Cruz I. Gutierrez-Juarez R. Lopez-Valdes H. E. (2023). Anti-inflammatory effects of flavonoids in common neurological disorders associated with aging. Int. J. Mol. Sci. 24, 4297. 10.3390/ijms24054297 36901731 PMC10001833

[B15] Muflihah Y. M. Gollavelli G. Ling Y. C. (2021). Correlation study of antioxidant activity with phenolic and flavonoid compounds in 12 Indonesian Indigenous herbs. Antioxidants (Basel) 10, 1530. 10.3390/antiox10101530 34679665 PMC8533117

[B16] Ozorowski M. Wicinski M. Kuzminski O. Wojciechowski P. Siedlecki Z. Sniegocki M. (2025). The effects of quercetin on vascular endothelium, inflammation, cardiovascular disease and lipid Metabolism-A review. Nutrients 17, 1579. 10.3390/nu17091579 40362888 PMC12073147

[B17] Sharifi-Rad J. Quispe C. Shaheen S. El Haouari M. Azzini E. Butnariu M. (2022). Flavonoids as potential anti-platelet aggregation agents: from biochemistry to health promoting abilities. Crit. Rev. Food Sci. Nutr. 62, 8045–8058. 10.1080/10408398.2021.1924612 33983094

[B18] Shen D. Labreche F. Wu C. Fan G. Li T. Dou J. (2022a). Ultrasound-assisted adsorption/desorption of jujube peel flavonoids using macroporous resins. Food Chem. 368, 130800. 10.1016/j.foodchem.2021.130800 34403997

[B19] Shen N. Wang T. Gan Q. Liu S. Wang L. Jin B. (2022b). Plant flavonoids: classification, distribution, biosynthesis, and antioxidant activity. Food Chem. 383, 132531. 10.1016/j.foodchem.2022.132531 35413752

[B20] Tao Y. Du M. Zhu M. Sun W. Zeng G. Xiong J. (2026). Anti-tumor effect of flavonoids isolated from Bidens Pilosa L. by regulating the activity of myeloid-derived suppressor cells within the tumor microenvironment in mice. J. Ethnopharmacol. 355, 120635. 10.1016/j.jep.2025.120635 40998135

[B21] Valko M. Leibfritz D. Moncol J. Cronin M. T. Mazur M. Telser J. (2007). Free radicals and antioxidants in normal physiological functions and human disease. Int. J. Biochem. Cell Biol. 39, 44–84. 10.1016/j.biocel.2006.07.001 16978905

[B22] Xu W. Yang J. Wu L. M. (2009). Cardioprotective effects of tanshinone IIA on myocardial ischemia injury in rats. Pharmazie 64, 332–336. 10.1691/ph.2009.8771 19530445

[B23] Yahia Y. Benabderrahim M. A. Tlili N. Bagues M. Nagaz K. (2020). Bioactive compounds, antioxidant and antimicrobial activities of extracts from different plant parts of two Ziziphus Mill. species. PLoS One 15, e0232599. 10.1371/journal.pone.0232599 32428000 PMC7236975

