# Supplementary material for: UPLC-MS/MS profiling, antioxidant and hemorheological effects of D101 resin-based purified flavonoids from Allium polyrhizum
Source: Front Pharmacol. 2026 Jun 3;17:1755360. doi: 10.3389/fphar.2026.1755360 (PMC13273041; doi:10.3389/fphar.2026.1755360)
Supplement: Supplementary file 1 [file Table1.docx]

**Supplementary Materials**

Table S1 Factors and levels of orthogonal experiment for total flavonoids purification

| Levels | Factors | | |
| --- | --- | --- | --- |
|  | Ethanol-water mixture eluent flow rates/(mL/min) | pH of sample solution | Concentration of ethanol-water mixture eluent /% |
| 1 | 3 | 4 | 60 |
| 2 | 4 | 5 | 70 |
| 3 | 5 | 6 | 80 |

Table S2 Recovery rates variance analysis of single factor experiment

| Variation source | Sum of squares | Degree of freedom | Mean square | F value | P value | Significance |
| --- | --- | --- | --- | --- | --- | --- |
| Sample flow rates | 334.528 | 4 | 83.632 | 0.581 | 0.684 | ns |
| pH of sample solution | 3129.052 | 4 | 782.263 | 6.001 | 0.00996 | ** |
| Concentration of ethanol-water mixture eluent | 19492.240 | 6 | 3248.707 | 27.314 | 5.839×10^-7^ | ** |
| Ethanol-water mixture eluent flow rates | 910.108 | 4 | 227.527 | 2.171 | 0.0146 | * |

Note: ns indicates no significant difference at *P*＞0.05 level, *indicates significant difference at *P*＜0.05 level, **indicates extremely significant difference at *P*＜0.01 level. The same below.

Table S3 Orthogonal experiment results and range analysis of extraction of total flavonoids

| NO. | Factors | | | Recovery rates/% |
| --- | --- | --- | --- | --- |
|  | A  Ethanol-water mixture eluent flow rates/(mL/min) | B  pH of sample solution | C  Concentration of ethanol-water mixture eluent/% |  |
| 1 | 1 | 1 | 1 | 80.804 |
| 2 | 1 | 2 | 2 | 85.249 |
| 3 | 1 | 3 | 3 | 83.043 |
| 4 | 2 | 1 | 2 | 84.767 |
| 5 | 2 | 2 | 3 | 94.715 |
| 6 | 2 | 3 | 1 | 72.457 |
| 7 | 3 | 1 | 3 | 86.344 |
| 8 | 3 | 2 | 1 | 79.944 |
| 9 | 3 | 3 | 2 | 77.097 |
| K1 | 249.096 | 251.915 | 233.205 |  |
| K2 | 251.939 | 259.908 | 247.113 |  |
| K3 | 243.375 | 232.597 | 264.102 |  |
| k1 | 83.032 | 83.972 | 77.735 |  |
| k2 | 83.980 | 86.636 | 82.371 |  |
| k3 | 81.128 | 77.532 | 88.034 |  |
| R | 2.852 | 9.104 | 10.299 |  |
| primary and secondary factors | | C＞B＞A | | |
| optimal solution | | A2B2C3 | | |

Table S4 Variance analysis of orthogonal experiment results

| Variation source | Square of deviance | Degree of freedom | *F*-ratio | *F* critical-values | Significance |
| --- | --- | --- | --- | --- | --- |
| A  Elution flow rate /(mL/min) | 12.652 | 2 | 0.125 | 5.140 | ns |
| B  pH of sample solution | 131.440 | 2 | 1.298 | 5.140 | ns |
| C  Concentration of ethanol-water mixture solution/% | 159.631 | 2 | 1.577 | 5.140 | ns |
| Error | 303.72 | 6 |  |  |  |

Table S5 Main flavonoid metabolites

| **Classification** | **Name** | **Relative molecular mass** | **Molecular formula** |
| --- | --- | --- | --- |
| Flavonoids | 6-C-MethylKaempferol-3-glucoside | 462.116 | C_22_H_22_O_11_ |
|  | Diosmetin-7-O-galactoside | 462.116 | C_22_H_22_O_11_ |
|  | Tamarixetin-3-O-(6''-malonyl)glucoside | 564.112 | C_25_H_24_O_15_ |
|  | Ayanin | 344.09 | C_18_H_16_O_7_ |
|  | Isorhamnetin-3-O-(6''-malonylglucoside) | 564.088 | C_25_H_24_O_15_ |
| Flavonols | Quercetin-3-O-galactoside (Hyperin)* | 464.095 | C_21_H_20_O_12_ |
|  | Quercetin-3-O-glucoside (Isoquercitrin)* | 464.095 | C_21_H_20_O_12_ |
|  | Isorhamnetin-7-O-glucoside (Brassicin)* | 478.111 | C_22_H_22_O_12_ |
|  | Quercetin-4'-O-glucoside (Spiraeoside)* | 464.095 | C_21_H_20_O_12_ |
|  | Quercetin-4′-O-glucuronide | 478.075 | C_21_H_18_O_13_ |
| Dihydroflavonols | \| Hesperetin-5-O-glucoside \| \| --- \| | \| 464.132 \| \| --- \| | \| C22H24O11 \| \| --- \| |
|  | Dihydrokaempferol-3-O-glucoside | 450.116 | C_21_H_22_O_11_ |
|  | Pinobanksin | 272.068 | C_15_H_12_O_5_ |
|  | Dihydromyricetin (Ampelopsin) | 320.053 | C_15_H_12_O_8_ |
|  | 3-O-Acetylpinobanksin | 314.079 | C_17_H_14_O_6_ |
| Anthocyanins | \| Cyanidin-3-O-(6''-O-malonyl)glucoside \| \| --- \| | \| 535.108 \| \| --- \| | C_24_H_23_O_14_ |
|  | Cyanidin-3-O-(3'',6''-O-dimalonyl)glucoside | 621.109 | C_27_H_25_O_17_ |
|  | Peonidin | 301.071 | C_16_H_13_O_6_ |
|  | Cyanidin-3-O-(2''-O-glucosyl)glucoside | 611.161 | C_27_H_31_O_16_ |
| Isoflavones | \| 6''-O-Malonylgenistin \| \| --- \| | \| 518.106 \| \| --- \| | \| C_24_H_22_O_13_ \| \| --- \| |
|  | 2'-Hydoxy,5-methoxyGenistein-4',7-O-diglucoside | 640.164 | C_28_H_32_O_17_ |
|  | Pratensein | 300.063 | C_16_H_12_O_6_ |
|  | 2'-Hydroxygenistein | 286.047 | C_15_H_10_O_6_ |
|  | Prunetin(5,4'-Dihydroxy-7-methoxyisoflavone) | 284.068 | C_16_H_12_O_5_ |
| Flavonoid C-glycosides | \| Apigenin-6-C-(2''-glucosyl)arabinoside \| \| --- \| | \| 564.148 \| \| --- \| | \| C_26_H_28_O_14_ \| \| --- \| |
|  | Isoschaftoside | 564.123 | C_26_H_28_O_14_ |
|  | Apigenin-6,8-di-C-arabinoside* | 534.137 | C_25_H_26_O_13_ |
|  | Apigenin-6-C-arabinoside-8-C-xyloside* | 534.137 | C_25_H_26_O_13_ |
|  | Luteolin-6-C-glucoside (Isoorientin) | 448.101 | C_21_H_20_O_11_ |
| Chalcones | \| Carthamone \| \| --- \| | \| 448.101 \| \| --- \| | \| C_21_H_20_O_11_ \| \| --- \| |
|  | 3,4,2',4',6'-Pentahydroxychalcone | 288.063 | C_15_H_12_O_6_ |
|  | Phloretin-4'-O-glucoside (Trilobatin) | 436.137 | C_21_H_24_O_10_ |
|  | Echinatin | 270.089 | C_16_H_14_O_4_ |
|  | Naringenin chalcone | 272.068 | C_15_H_12_O_5_ |
| Dihydroflavonoids | \| Eriodictyol-7-O-glucoside \| \| --- \| | \| 450.116 \| \| --- \| | \| C_21_H_22_O_11_ \| \| --- \| |
|  | Eriodictyol (5,7,3',4'-Tetrahydroxyflavanone) | 288.063 | C_15_H_12_O_6_ |
|  | Homoeriodictyol | 302.079 | C_16_H_14_O_6_ |
|  | Butin | 272.068 | C_15_H_12_O_5_ |
|  | Hesperetin | 302.079 | C_16_H_14_O_6_ |
| Flavanols | \| 4'-Hydroxy-5,7-dimethoxyflavanone \| \| --- \| | \| 300.1 \| \| --- \| | \| C_17_H_16_O_5_ \| \| --- \| |
|  | Gallocatechin-(4α→8)-gallocatechin | 610.132 | C_30_H_26_O_14_ |
|  | Naringenin-7-O-(6''-malonyl)glucoside | 520.122 | C_24_H_24_O_13_ |
